# Rare origin of the sinoatrial node artery: an anatomic report and a brief review of the literature

**DOI:** 10.1007/s12565-024-00779-1

**Published:** 2024-05-24

**Authors:** Filipe F. Pinto, M. Dulce Madeira, Pedro A. Pereira

**Affiliations:** 1 Unit of Anatomy, Department of Biomedicine, Faculty of Medicine, University of Porto, Alameda Prof. Hernâni Monteiro, 4200-319 Porto, Portugal; 2 NeuroGen Research Group, Center for Health Technology and Services Research (CINTESIS), Rua Dr. Plácido da Costa, 4200-450 Porto, Portugal; 3 CINTESIS@RISE, Faculty of Medicine, University of Porto, Alameda Prof. Hernâni Monteiro, 4200-319 Porto, Portugal

**Keywords:** Anatomy, Anatomical variations, Clinical relevance, Human cadaveric dissection, Sinoatrial node artery

## Abstract

Several studies reported anatomical variations in the sinoatrial node artery (SANa). Here, we report a rare variation in the origin of the SANa on a human adult male cadaver. During dissection, we identified the SANa originating from a large atrial branch of the right coronary artery (RCA). This branch originates at the level of the inferior border of the heart and courses upwards. The initial part of this vessel is tortuous, and then it follows a straight path parallel to the RCA along the anterior surface of the right atrium. After this part, the artery curves posteriorly and to the left until it reaches the lower border of the right auricle, where it closely approaches the RCA. Finally, the artery runs posteriorly and to the right to follow a course along the medial wall of the right auricle and right atrium to reach a location close to the region of the junction of the superior vena cava and right atrium, where it follows its path buried in the myocardium. After perforating the myocardium, this vessel gives rise to branches that are distributed to both atria in addition to the SANa. The SANa runs to the sinoatrial node in a precaval (anterior to the superior vena cava) course. We also tried to characterize the vessels radiologically. The knowledge of the anatomical variations of the SANa is of the utmost importance for cardiologists and heart surgeons to better understand cardiac disease and accurately plan and execute cardiac interventions and surgical procedures.

## Introduction

The sinoatrial node is located in the wall of the right atrium, in the upper part of the sulcus terminalis at the junction of the superior vena cava and the right atrium, and functions as the pacemaker of the heart (Futami et al. 2003; Kawashima and Sasaki 2003; Standring 2021). The sinoatrial node artery (SANa), an artery whose origin, number, size, and course are reported to be variable, irrigates, among other cardiac structures, the sinoatrial node (Boulemden et al. 2019; Esrailian et al. 2023; Nerantzis et al. 2021; Ortale et al. 2006; Pejković et al. 2008; Shimotakahara et al. 2014; Vikse et al. 2016). As a result, it is clinically relevant, in addition to being anatomically significant, given that it is used as a landmark for the identification of the sinoatrial node (Kawashima and Sasaki 2003; Standring 2021; Vikse et al. 2016). In a meta-analysis study published in 2016, Vikse and collaborators concluded that the SANa is more commonly a single vessel, originating from the right coronary artery (RCA), and following more frequently a retrocaval route (Vikse et al. 2016). When the SANa emerges from the RCA, it tends to arise from its proximal segment (Hutchinson 1978; Sow et al. 1996; Vikse et al. 2016). Nevertheless, there are studies reporting an uncommon origin of the SANa from the more distal part of the RCA or even from its more distal branches (Futami et al. 2003; Hutchinson 1978; Kara et al. 2014; Kyriakidis et al. 1988; Lotfian et al. 2022; Nerantzis et al. 2011).

An adequate understanding of the normal vascular anatomy and of the anatomical variations is vital in several medical specialties so as to accurately perform both a broad range of diagnoses and innumerous medical procedures (Di Candia et al. 2021; Esrailian et al. 2023; Nerantzis 2021; Nerantzis et al. 2011, 2021; Pinho et al. 2022; Sow et al. 1996; Standring 2021; Vikse et al. 2016). However, despite their importance in effective diagnosis and treatment, such variations are often overlooked in medical school curricula and clinical practice. Thus, in the current study, we describe a rare anatomical variation of the origin of the SANa through dissection and radiological approaches.

## Case report

A formalin-embalmed adult male human Portuguese cadaver (Caucasian, 82 years old) was subjected to routine dissection of the thoracic cavity. The cadaver derived from a body donation with informed consent, written and signed by the donor himself (Portuguese Decree-Law Nº 274/99). The authors hereby confirm that every effort was made to comply with all local and international ethical guidelines and laws concerning the use of human cadaveric donors in anatomical research. The complete medical history of the donor was not available. The first part of the dissection was carried out with the cadaver placed in a supine position, and all the dissection procedures were carefully performed by using appropriate dissection techniques and proper dissection tools aiming to not disturb the normal anatomy of the thoracic cavity (Loukas et al. 2019; Standring 2021). Specifically, regarding the case report herein presented, the pericardium, the heart and the juxtacardiac parts of its great vessels, along with the coronary arteries and its branches and the cardiac veins were dissected.

**Fig. 1:**
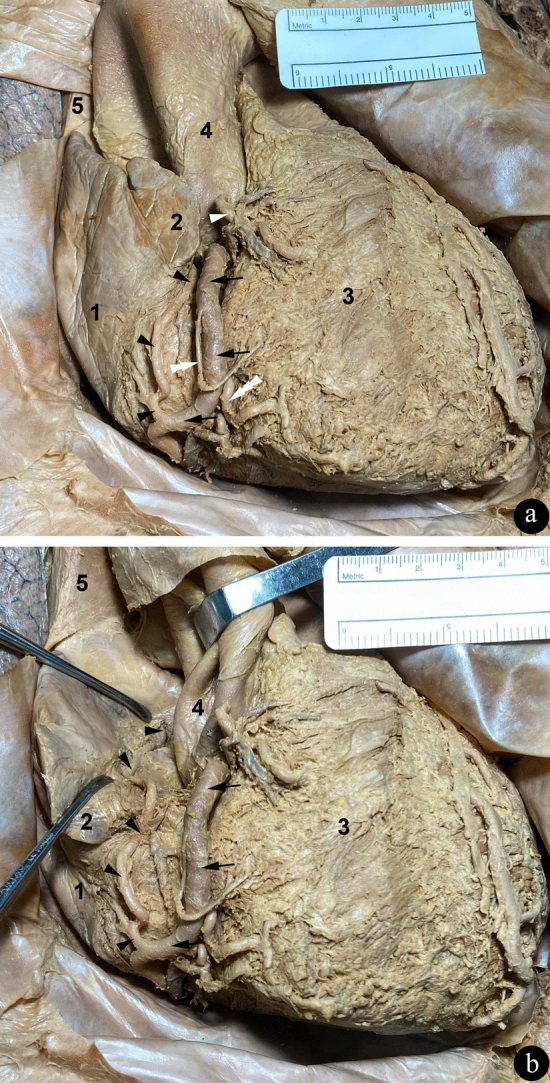
Anterior views of the heart and juxtacardiac parts of its great vessels. The pericardium has been partially retracted (**a**, **b**). **a** The cardiac and vascular dissected structures are in their proper positions. **b** The ascending aorta was retracted medially and the right atrium and right auricle were retracted laterally in order to observe the course of the atrial branch of the RCA until it perforates the myocardium. Black arrows indicate the course of the RCA and black arrowheads indicate the course of its atrial branch. Other branches of the RCA are also labeled (**a**): right conal artery (single white arrowhead), right ventricular artery (double white arrowhead) and right marginal artery (triple white arrowhead). Indication of cardiac chambers and great vessels: 1, right atrium; 2, right auricle; 3, right ventricle; 4, ascending aorta; 5, superior vena cava

During the dissection of the initial part of the RCA, we tried to find the SANa bearing in mind that it more commonly arises from this part of the RCA. In spite of that, we did not detect the SANa in this location and we continued with the dissection of the RCA. At the level of the inferior border (acute margin) of the heart, the described RCA follows an unusual and almost horizontal trajectory to the right before curving around the inferior (acute) border of the heart into the inferior part of the atrioventricular (coronary) sulcus, where it continues toward the cardiac crux. Originating from this almost horizontal segment of the RCA, we identified a large caliber branch arising from its anterosuperior aspect and running upwards (Fig. 1a). Afterward, we dissected this branch and pursued its course. First, the artery follows a tortuous path and then it runs approximately parallel to the coronary sulcus and RCA along the anterior surface of the right atrium. More superiorly, it bends posteriorly and to the left until it reaches the lower border of the right auricle. In this part of its trajectory, the artery is located close to and inferiorly and to the right of the RCA. After that, the vessel continues posteriorly and to the right to follow a route along the medial surface of the right auricle and right atrium to reach a point in the vicinity of the region of the junction of the right atrium and superior vena cava where it enters the myocardium of the right atrium (Fig. 1b), thus initially appearing to be the SANa.

**Fig. 2:**
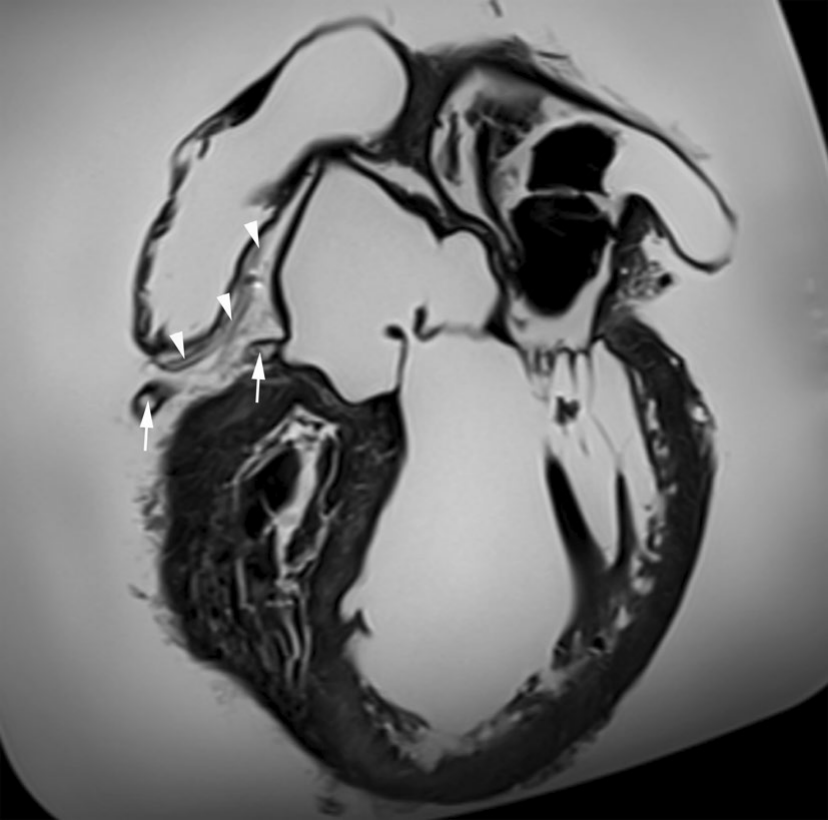
Magnetic resonance imaging (from T2-weighted sequences) showing part of the course of the RCA and its atrial branch. The white arrows indicate the RCA and the white arrowheads the atrial branch of the RCA

Then, for the purpose of continuing the dissection of the RCA branch in a more accessible way, we decided to carefully remove the heart from the cadaver’s thorax by sectioning the great vessels, i.e., the superior vena cava, the inferior vena cava, the pulmonary veins, the pulmonary trunk, and the aorta. Furthermore, before continuing the dissection, and taking advantage of the fact that the heart was isolated, we tried to visualize the atrial branch’s intramyocardial course and its possible branches, by using an accurate imagiological method, i.e., magnetic resonance imaging. To achieve this, the heart was embedded in liquid commercial gelatin and was then refrigerated at 4 °C to solidify. Then, the magnetic resonance images were acquired in a 3 T scanner (Siemens Magnetom Vida) using mainly T2-weighted sequences (Fig. 2). However, after careful analysis of the obtained images, it was not possible to achieve the proposed goal.

**Fig. 3:**
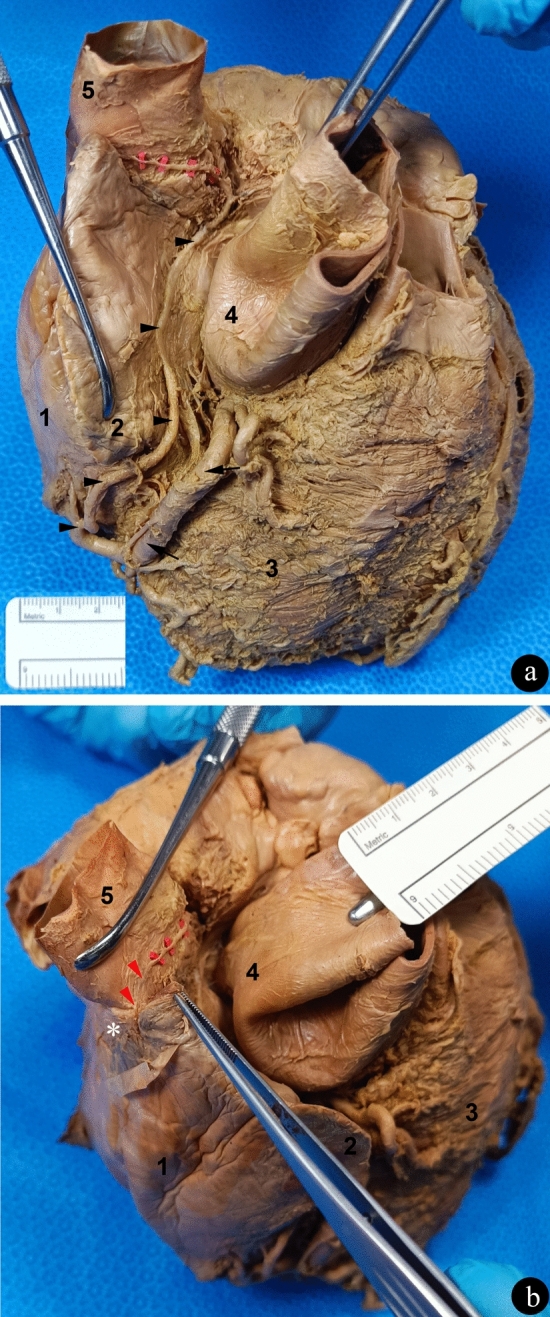
Views of the isolated heart and the juxtacardiac parts of its great vessels. **a** The ascending aorta was retracted to the left and the right atrium and the right auricle to the right to observe the course of the atrial branch of the RCA and the SANa. The black arrows indicate the RCA, the black arrowheads indicate the atrial branch of the RCA and the red rectangles mark the SANa. **b** Zoomed view of the SANa (red rectangles and red arrowheads) and the sinoatrial node (white *). Indication of cardiac chambers and great vessels: 1, right atrium; 2, right auricle; 3, right ventricle; 4, ascending aorta; 5, superior vena cava

Afterward, we removed part of the myocardium to expose the remaining part of the artery. After perforating the myocardium, we noticed that this vessel gives rise to branches that are distributed to both atria in addition to the SANa, thus confirming that it is indeed appropriate to name the RCA large caliber branch that originates at the inferior border of the heart as an atrial branch, as we have been doing so far (Standring 2021). Actually, we identified the SANa as a vessel that runs to the sinoatrial node in a precaval (anterior to the superior vena cava) course (Fig. 3a and b). In detail, the SANa initially curves superiorly and to the right, and then it follows a straight course to the right between the superior portion of the right auricle and the inferior extremity of the superior vena cava, following a precaval trajectory. It ends running slightly inferiorly to the region where the sinoatrial node is commonly described. Considering the subepicardial location, shape and consistency of the tissue that we also approached by dissection in that region supplied by SANa (He et al. 1991; Nooma et al. 2020; Standring 2021), we believe that we may have identified the sinoatrial node (Fig. 3b).

During dissection, we took several morphological measurements. The length of the RCA from its origin to the origin of its atrial branch that originates the SANa, the length of the mentioned atrial branch from its origin to the point where it perforates the myocardium and to the point of origin of the SANa, and the length of the SANa from its origin to the point where it perforates the sinoatrial node were measured using a non-elastic wire that was placed for the purpose of following the course of the vessels. The wire was subsequently placed in a straight line on a table and measured by using a digital caliper. The same digital caliper was used to measure the diameter of the above-mentioned atrial branch at its origin, at the site where it perforates the myocardium, and at the site where it originates the SANa, as well as the diameter of SANa at its origin. By using a goniometer, we also measured the angle of origin of the atrial branch from the RCA as well as the angle of origin of the SANa from its parent trunk.

In regard to the morphometric data, we found that the length of the RCA from its origin in the ascending aorta to the origin of the atrial branch was 71.23 mm. The length of this atrial branch from its origin to the site where it perforates the myocardium was 100.14 mm, and from this point until the origin of the SANa it was 9.26 mm. The length of the SANa from its origin to the site where it perforates sinoatrial node was 32.35 mm. The diameter of the atrial branch at its origin, where it enters the myocardium and where it originates to the SANa was 2.35 mm, 1.90 mm, and 1.65 mm, respectively. The diameter of SANa at its origin was 1.10 mm. The angle of origin of the atrial branch from RCA was an obtuse angle (approximately 95º), as was the angle origin of the SANa from its parent trunk (approximately 120º).

Moreover, in the segment of the RCA between its emergence from the ascending aorta and the inferior (acute) border of the heart, we did not identify any anterior atrial branches. However, the larger RCA branch originating near the inferior border that we dissected gives rise to several branches that reach the right atrium as well as the left atrium (Figs. 1 and 3a). Regarding other branches of the RCA (Fig. 1a), we highlight the right conal artery that courses anteroinferiorly over the conus arteriosus (infundibulum) and over the superior aspect of the right ventricle (Loukas et al. 2016; Standring 2021) as well as a right ventricular branch and the right marginal artery (Standring 2021).

## Discussion

Considering the complex embryology of the coronary arteries, it is expected that changes in development can lead to coronary artery anomalies and variations (Loukas et al. 2009; Tomanek and Angelini 2019). Here, we presented a rare case of variation in the origin of the SANa. Although we did not perform a histological study, the route and distribution seen macroscopically enabled us to conclude that in fact we identified the SANa. In our case, the SANa has an uncommon origin from the sole atrial branch of the segment of the RCA between its emergence from the ascending aorta and the inferior border of the heart.

In our study, the atrial branch that gives rise to the SANa originates just before the RCA curves around the inferior border of the heart to the inferior part of the atrioventricular sulcus. We observed that the length of the RCA measured from the aorta to the origin of that atrial branch was 71.23 mm, and that the length of its atrial branch to the origin of the SANa was 109.40 mm. This shows that the trajectory from the ostium of the RCA to the emergence of the SANa is relatively long (180.63 mm). In a meta-analysis, Vikse and collaborators analyzed 18 studies in which the pooled mean distance between the origin of the SANa and the ostia of the RCA was 16.306 mm (Vikse et al. 2016). Therefore, contrarily to what happens in the majority of cases, in which the SANa originates from the initial part of the RCA, the artery that we detail in the present study has a considerable distant origin from the emergence of the RCA from the ascending aorta. Notably, there are studies reporting sinoatrial node arteries arising from the RCA at a maximal distance of 62 mm (Ozturk et al. 2011) and 84.3 mm (Cezlan et al. 2012) from the RCA ostium, distances that are very similar to the distance (71.23 mm) of the origin of the atrial branch that originates the SANa to the ostium of the RCA. Therefore, although the SANa herein described does not directly originate from RCA, it emerges indirectly, through the RCA atrial branch, near the inferior border of the heart. Interestingly, there are studies that report cases in which the SANa arises at or near the inferior border of the heart (Hutchinson 1978; Kyriakidis et al. 1988). In one of the aforementioned studies, it was observed in 1 out of 309 (0.32%) patients studied (Kyriakidis et al. 1988), and in the other it was found in 6 out of 40 (15%) hearts studied (Hutchinson 1978). Importantly, from the 309 patients studied by Kyriakidis and collaborators (1988), only 2 showed atherosclerotic plaques and one of them was the SANa that emerged near the origin of the acute marginal artery. Similarly, Ozturk and colleagues discovered an atherosclerotic plaque in the SANa in 3 cases out of 251 patients (Ozturk et al. 2011). In this context, it is important to emphasize that it was recently observed in a study carried out on 59 adult hearts (average age of subjects, 70.6 ± 14.5 years) that, independent of the SANa origin, calcification rarely occurs in this artery in old age (Tohno et al. 2018).

The course of the SANa in relation to the superior vena cava can be variable (Vikse et al. 2016). In fact, the SANa artery can run anterior to the superior vena cava (precaval), posterior to the superior vena cava (retrocaval) or in a combination of both (pericaval). Even though it is found that the SANa follows more frequently a retrocaval route (Vikse et al. 2016), the SANa we identified follows a precaval course.

Concerning the diameter of the SANa, in a meta-analysis study published in 2016, Vikse and collaborators reported that at its origin, the pooled mean diameter of the SANa originating from the RCA in the European population was 1.324 (0.884–1.764) mm (Vikse et al. 2016). The measure obtained in our study regarding this parameter (1.10 mm) falls within the scope of values detailed in the above-mentioned study.

The angle of origin of the atrial branch from the RCA, was obtuse (approximately 95º) as was the angle of origin of the SANa from the mentioned atrial branch (approximately 120º). It was previously verified that in most hearts (48%) the SANa originates from the parent trunk at an obtuse angle (Pare et al. 2017). The angle of origin of SANa is clearly an anatomic and clinically relevant issue concerning the blood circulation in the arteries since the risk of thrombotic obstruction increases with the acuteness of its branching pattern from its parent trunk (Pare et al. 2017).

In conclusion, in the present study, we describe a rare variation of the SANa found during a routine cadaveric dissection. The knowledge of the anatomical variations of the SANa, even if it is one of its rarest variations, is valuable for anatomists and of great clinical significance for cardiac surgeons and interventional cardiologists in carrying out diagnoses as well as in planning and executing invasive cardiac procedures and surgical interventions.

## Data Availability

The data that support the findings of this study are available from the corresponding author, upon reasonable request.
